# Global progress in abortion law reform: a comparative legal analysis since the International Conference on Population and Development (1994–2023)

**DOI:** 10.1080/26410397.2025.2499324

**Published:** 2025-04-28

**Authors:** Katy Mayall, Laurenne Ajayi, Caitlin Gruer

**Affiliations:** a Director of Strategic Initiatives, Center for Reproductive Rights, New York, NY, USA.; b Legal Fellow, Center for Reproductive Rights, Nairobi, Kenya; c Program Manager, Center for Reproductive Rights, New York, NY, USA

**Keywords:** abortion, human rights, reproductive, reproductive rights

## Abstract

As 2024 marked the 30th anniversary of the International Conference on Population and Development, which recognised unsafe abortion as a human rights and public health imperative, it is an apt time to assess global progress on abortion law reform. By mapping changes to abortion laws for 199 nations and semi-autonomous territories from 1994 to 2023 and coupling this with population data, this article demonstrates that the past three decades have been marked by an overwhelming trend towards the liberalisation of abortion laws across all regions, resulting in over 825 million women of reproductive age living under expanded grounds for legal abortion. Notably, a potential sea change has occurred in abortion law reform in the past five years, with countries increasingly liberalising their laws to permit abortion on request instead of adopting more incremental approaches. More countries have reformed their laws to permit abortion on request in the past five years than in the 25 preceding years. Yet, significant disparities continue to exist across regions. Countries banning abortion altogether or narrowly permitting abortion when the pregnant person’s life is at risk are concentrated in Africa, Asia, and Latin America, including in resource-scarce contexts where abortion seekers are doubly disadvantaged by restrictive laws and limited access to healthcare, including post-abortion care. It is critical that law and policymakers and public health authorities recognise that restrictive abortion laws are out of step with global norms and support progress towards the liberalisation of abortion laws, particularly towards permitting abortion on request.

## Plain language summary

The landmark International Conference on Population and Development took place in 1994. Since then, there has been an overwhelming trend towards liberalising abortion laws across all regions of the globe. Over 60 countries worldwide have liberalised their abortion laws. These liberalisations have resulted in approximately 825 million women of reproductive age living under expanded grounds for legal abortion than in 1994. During this time, only five countries have removed legal grounds for abortion or reduced the time limit for abortion on request. In total, these regressions impact 92 million women of reproductive age. The trend towards liberalisation marks a significant shift in the proportion of countries globally with restrictive abortion laws. There has been a significant movement away from the complete prohibition of abortion. More countries are permitting abortion on request. But such progress is not uniform and significant disparities continue to exist. 25% of women of reproductive age still live in countries that ban abortion altogether or narrowly permit it when the pregnant person’s life is at risk. This population is concentrated in Africa, Asia, and Latin.

## Résumé

Alors que 1994 a marqué le 30^e^ anniversaire de la Conférence internationale sur la population et le développement, qui a reconnu l’avortement à risque comme un impératif des droits de l’homme et de la santé publique, le moment est bien choisi pour évaluer les progrès accomplis dans le monde en matière de réforme du droit à l’avortement. En recensant les changements apportés aux lois sur l’avortement dans 199 nations et territoires semi-autonomes entre 1994 et 2023 et en les croisant avec les données démographiques, cet article démontre que les trois décennies passées ont été marquées par une tendance massive à la libéralisation des lois sur l’avortement dans l’ensemble des régions, ce qui a permis à plus de 825 millions de femmes en âge de procréer de vivre dans des environnements acceptant des motifs élargis d’avortement légal. Il est à noter qu’un changement radical s’est produit dans la réforme du droit à l’avortement ces cinq dernières années, les pays libéralisant de plus en plus leurs lois pour autoriser l’avortement sur demande au lieu d’adopter des approches plus graduelles. Davantage de pays ont réformé leurs lois pour permettre l’avortement sur demande ces cinq dernières années que pendant le quart de siècle précédent. Pourtant, de profondes disparités persistent entre les régions. Les pays interdisant totalement l’avortement ou l’autorisant de manière restrictive lorsque la vie de la personne enceinte est en danger se concentrent en Afrique, en Asie et en Amérique latine, notamment dans des contextes de pénurie de ressources où les personnes souhaitant avorter sont doublement défavorisées par des lois restrictives et un accès limité aux soins de santé, y compris aux soins post-avortement. Il est essentiel que les législateurs, les décideurs et les autorités de santé publique reconnaissent que les lois restrictives sur l’avortement sont en décalage avec les normes mondiales et soutiennent les progrès vers la libéralisation du droit à l’avortement, en particulier en vue d’autoriser l’avortement sur demande.

## Resumen

Dado que en 1994 se conmemoró el 30^o^ aniversario de la Conferencia Internacional sobre la Población y el Desarrollo, que reconoció el aborto inseguro como un imperativo de derechos humanos y salud pública, es un momento oportuno para evaluar el progreso mundial en la reforma de las leyes de aborto. Al mapear los cambios a las leyes de aborto de 199 naciones y territorios semiautónomos entre 1994 y 2023 y combinar esto con los datos de población, este artículo demuestra que las últimas tres décadas estuvieron marcadas por una tendencia abrumadora hacia la liberalización de las leyes de aborto en todas las regiones, por lo cual más de 825 millones de mujeres en edad reproductiva viven en lugares con causales ampliadas de aborto legal. Notablemente, en los últimos cinco años se ha producido un posible cambio radical en la reforma de las leyes de aborto, ya que cada vez más países liberalizan sus leyes para permitir el aborto a petición, en vez de adoptar enfoques más incrementales. Más países han reformado sus leyes para permitir el aborto a petición en los últimos cinco años que en los 25 años anteriores. Sin embargo, continúan existiendo disparidades significativas entre regiones. Los países con una prohibición absoluta del aborto o que permiten el aborto solo cuando la vida de la persona embarazada corre peligro están concentrados en África, Asia y América Latina, en contextos con escasos recursos donde las personas que buscan un aborto están doblemente desfavorecidas debido a las leyes restrictivas y al acceso limitado a servicios de salud, tales como la atención postaborto. Es imperativo que los legisladores, formuladores de políticas y autoridades de salud pública reconozcan que las leyes de aborto restrictivas están fuera de sintonía con las normas mundiales y que apoyen el progreso hacia la liberalización de las leyes de aborto, en particular hacia permitir el aborto a petición.

## Introduction

The 30th anniversary of the International Conference on Population and Development (ICPD) this past year marked an important moment to take stock of the progress and setbacks seen in the pursuit of abortion rights across the globe, as an indicator of both reproductive health and gender equity. Laws denying people the right to decide whether to carry a pregnancy to term directly impact individuals’ educational attainment, economic opportunities, and ability to participate in public and political life, disproportionately impacting women due to both their reproductive capacities and historical role as caretakers. This article analyses the extent to which countries around the world have reformed their abortion laws in the 30 years since ICPD, examining progress and regression at both the global and regional levels. It then discusses critical global developments since ICPD and the importance of ensuring that reproductive rights, including abortion, are and continue to be fully recognised as fundamental human rights.

In 1994, ICPD convened governments from around the world to agree on a global agenda for population and development. The resulting Programme of Action (PoA) marked a paradigm shift in population policy, moving away from population control to a focus on reproductive health, rights, and autonomy.^1,2^ The PoA’s explicit inclusion of abortion as part of reproductive health was also a welcome and significant departure from previous political agreements, albeit the new language was limited in scope. The PoA urged governments to “strengthen their commitment to women’s health to deal with the impact of unsafe abortion as a major public health concern,” affirmed that “where abortion is not against the law, such abortion should be safe,” and noted that in all cases, “women should have access to quality abortion services for the management of complications arising from abortion.”^3^

Despite entrenched political resistance to abortion rights in many contexts, the harmful effects of restrictive abortion laws on individuals’ lives, health, and well-being are well-documented. The World Health Organization (WHO) recognises that restrictive abortion laws do not eliminate the need for abortion, but instead push it underground, forcing pregnant people to seek clandestine procedures which are more likely to be unsafe.^4^ This has devastating consequences; between 4.7% and 13.2% of all maternal deaths are attributed to unsafe abortion, equating to between 13,865 and 38,940 deaths annually.^5^ Historically marginalised and underserved populations bear the brunt of these impacts: the WHO reports that increasingly, unsafe abortions are concentrated in developing nations, representing 97% of unsafe abortions globally.^5^ Nearly all deaths related to unsafe abortion could be averted through the provision of safe, quality abortion care.^5^ Yet, in almost every country worldwide – even those with relatively liberal abortion laws – abortion continues to be regulated through criminal law instead of being recognised and treated as a form of healthcare.

## Methodology

This paper maps the abortion laws of 199 countries, semi-autonomous regions, and jurisdictions of special status and the changes to these laws over the past 30 years. This includes independent states, semi-autonomous regions, territories, and jurisdictions of special status whose populations exceed one million. This total reflects the 193 United Nations member states, plus 2 nonmember states (Kosovo and the State of Palestine) and 4 territories or other administrative jurisdictions (Hong Kong, Northern Ireland, Puerto Rico, and Taiwan). The legal status of each country is considered through a strict reading of the black letter law; while there may be supplementary guidelines and policies in place in some contexts, they are excluded from consideration unless they have the force of law.

This mapping uses a classification system developed by the Center for Reproductive Rights which distributes countries’ abortion laws into six mutually exclusive categories: (I) abortion permitted on request (gestational limits vary); (II) abortion permitted on broad social or economic grounds; (III) abortion permitted on health grounds (including risk to physical and mental health); (IV) abortion permitted to save the pregnant person’s life; (V) abortion prohibited altogether; and (VI) laws vary at state level (federal systems). For laws in Categories II through IV, we have also mapped three common enumerated grounds under which abortion is legal (cases of rape, incest, and fetal diagnoses). The Center for Reproductive Rights has been using a version of this framework to analyse global abortion laws since 1998. We aggregated the number of women of reproductive age (15–49) from all the countries included in our analysis to calculate both the absolute number and the percentage of women of reproductive age who live in countries that fall into each of the six categories of abortion laws. The use of “women of reproductive age” as opposed to “people with the capacity to become pregnant” reflects a limitation in how countries gather population data and is not meant to obscure the need for abortion care among trans and non-binary persons. The findings were also disaggregated using the United Nations Population Division’s regional classifications for Africa, Asia, Europe, Latin America and the Caribbean (LAC), Northern America, and Oceania.

This research also explores the trends in abortion laws over the past 30 years, utilising the Center for Reproductive Rights’ historical data categorising countries’ abortion laws since 1994.^4,6,7^ By comparing each country’s current categorisation to its previous categorisations, we were able to map both liberalisations and retrogressions. For the purposes of this analysis, we defined liberalisation as the expansion of the grounds under which abortion is legal or the extension of the gestational limit in countries where abortion is broadly legal (Categories I and II), and defined retrogression as the removal of legal grounds for abortion or the lowering of the gestational limit in countries where abortion is broadly legal. To quantify the impact of these liberalisations and retrogressions, we utilised the current population data from the United Nations Population Division to estimate the number of women of reproductive age living today who have been impacted by changes to abortion laws in each region.^8^ This analysis provides a framework for understanding the scale of the impact that such liberalisations and retrogressions have had on individuals’ lives.

There are three authors of this study. One author self-identifies as a cis white American woman, another self-identifies as a cis mixed British-Nigerian woman, and the third self-identifies as a cis white British-American woman. All currently live and work in the US or UK, though each has experience living and working internationally, including in the global majority countries.

## Results

Of the 199 countries, semi-autonomous regions, and jurisdictions of special status included in this review, 75 countries, accounting for 34% of women of reproductive age (WRA), permit abortion on request (Category I). Twelve countries (23% of WRA) permit abortion on broad social or economic grounds (Category II), while 47 countries (12% of WRA) permit abortion to preserve the person’s health (Category III). Forty-five countries (20% of WRA) permit abortion to save the life of the pregnant person (Category IV) and 18 countries (5% of WRA) prohibit abortion altogether (Category V). Finally, there are two countries (6% of WRA) where the abortion laws vary significantly at the sub-national level: Mexico and the United States (Table 1).

**Table 1.** Distribution of countries, semi-autonomous regions, and jurisdictions of special status by abortion legality category

Abortion legality	Percentage of WRA globally	Country, semi-autonomous region, and jurisdiction of special status
Category I – On request (gestational limits vary)	34%	Albania, Argentina, Armenia, Australia, Austria, Azerbaijan, Belarus, Belgium, Benin, Bosnia & Herzegovina, Bulgaria, Cabo Verde, Cambodia, Canada, China, Colombia, Croatia, Cuba, Cyprus, Czechia, Democratic People’s Republic of Korea, Denmark, Equatorial Guinea, Estonia, Finland, France, Georgia, Germany, Greece, Guinea-Bissau, Guyana, Hungary, Iceland, Ireland, Italy, Kazakhstan, Kosovo, Kyrgyzstan, Latvia, Lithuania, Luxembourg, Maldives, Moldova, Mongolia, Montenegro, Mozambique, Nepal, Netherlands, New Zealand, North Macedonia, Northern Ireland, Norway, Portugal, Republic of Korea, Romania, Russian Federation, San Marino, São Tomé & Príncipe, Serbia, Singapore, Slovak Republic, Slovenia, South Africa, Spain, Sweden, Switzerland, Tajikistan, Thailand, Tunisia, Türkiye, Turkmenistan, Ukraine, Uruguay, Uzbekistan, Viet Nam
Category II – Broad social or economic grounds	23%	Barbados, Belize, Ethiopia, Fiji, Great Britain, Hong Kong, India, Japan, Rwanda, Saint Vincent & the Grenadines, Taiwan, Zambia
Category III – To preserve health	12%	Algeria, Angola, Bahamas, Bolivia, Botswana, Burkina Faso, Burundi, Cameroon, Central African Republic, Chad, Comoros, Costa Rica, Democratic Republic of the Congo, Djibouti, Ecuador, Eritrea, Eswatini (formerly Swaziland), Ghana, Grenada, Guinea, Israel, Jordan, Kenya, Kuwait, Lesotho, Liberia, Liechtenstein, Malaysia, Mauritius, Monaco, Morocco, Namibia, Nauru, Niger, Pakistan, Peru, Poland, Puerto Rico, Qatar, Saint Lucia, Samoa, Saudi Arabia, Seychelles, Togo, Trinidad & Tobago, Vanuatu, Zimbabwe
Category IV – To save a person’s life	20%	Afghanistan, Antigua & Barbuda, Bahrain, Bangladesh, Bhutan, Brazil, Brunei Darussalam, Chile, Côte d'Ivoire, Dominica, Gabon, Gambia, Guatemala, Indonesia, Iran, Kiribati, Lebanon, Libya, Malawi, Mali, Malta, Marshall Islands, Mauritania, Micronesia, Myanmar, Nigeria, Oman, Palestine, Panama, Papua New Guinea, Paraguay, Saint Kitts & Nevis, Solomon Islands, Somalia, South Sudan, Sri Lanka, Sudan, Syrian Arab Republic, United Republic of Tanzania, Timor-Leste, Tuvalu, Uganda, United Arab Emirates, Venezuela, Yemen
Category V – Prohibited altogether	5%	Andorra, Congo, Dominican Republic, Egypt, El Salvador, Haiti, Honduras, Iraq, Jamaica, Lao People’s Democratic Republic, Madagascar, Nicaragua, Palau, Philippines, Senegal, Sierra Leone, Suriname, Tonga
Category VI – Varies at the state level	6%	Mexico, United States of America

### Global trends

Since 1994, there has been an overwhelming global trend towards liberalisation, with over 60 countries liberalising their abortion laws. As a result, over 825 million WRA are living under expanded grounds for legal abortion as compared to 30 years ago.

These liberalisations mark a significant shift in the proportion of countries globally with restrictive abortion laws. In 1994, 40 countries prohibited abortion under all circumstances; at the end of 2023, just 18 countries remained in this category. Similarly, these liberalisations have shifted the proportion of countries with the most liberal abortion laws. In 1994, there were 47 countries that permitted abortion on request; by the end of 2023, this had increased to 75 countries.

Of the countries that have liberalised their abortion laws, nearly one-half (27 countries) have reformed their laws to permit abortion on request. Notably, many of these reforms are relatively recent: in the five-year period from 2019 to 2023, 12 countries liberalised their laws to permit abortion on request. By contrast, in the 25-year period from 1994 to 2018, almost the same number of countries (15) reformed their laws to permit abortion on request.

In addition, there has been significant movement away from the complete prohibition of abortion. Since 1994, 22 countries have overturned absolute bans on abortion, the majority of which are in Africa (13 countries). Globally, these reforms have varied from relatively minor developments, such as the seven countries that reformed their laws to narrowly permit abortion when the pregnant person's life is at risk, to more substantial overhauls in which countries legalised abortion under a much broader set of circumstances. For example, in 2012, Somalia overturned an absolute prohibition to permit abortion in order to save the life of a pregnant person, moving it one category across the spectrum. Meanwhile, in 2002, Nepal amended its blanket ban on abortion to permit abortion on request up to 12 weeks gestation and on specific grounds thereafter. This reform took Nepal entirely across the spectrum, from Category V to Category I.

**Figure 1.**
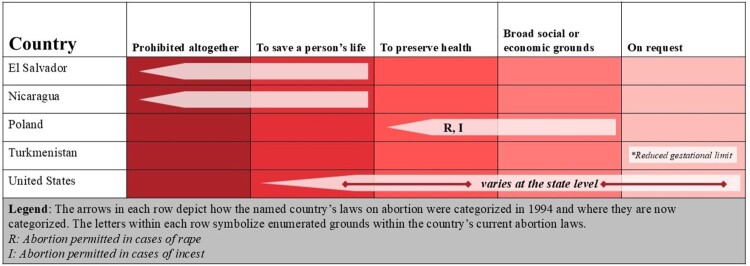
Retrogressions in countries’ abortion laws from 1994 to 2023

During this same period, only five countries have either removed legal grounds for abortion or reduced their gestational limits where abortion is broadly permitted. Four countries – the United States, El Salvador, Nicaragua, and Poland – have removed legal grounds for abortion (See Figure 1). Two of these countries, El Salvador and Nicaragua, removed laws permitting abortion when the pregnant person’s life was at risk in order to prohibit abortion altogether. Poland is unique in having its Constitutional Court twice invalidate specific legal grounds for abortion, first in 1997 (when it invalidated broad social and economic grounds) and again in 2020 (when it invalidated grounds based on fetal diagnoses). In the US, the overturning of *Roe v. Wade* in 2022 has resulted in abortion laws which vary significantly at the state level. In addition, in 2022 one country, Turkmenistan reduced its gestational limit for abortion on request from 12 to 5 weeks. In total, these five regressions impact 92 million WRA.

### Trends by region

The legality of abortion and the pace of liberalisations varies significantly across regions. As seen in Table 2, whereas the overwhelming majority of WRA in Asia (73%), Europe (94%), and Oceania (71%) live where abortion is legal on request or on broad social or economic grounds (Categories I and II), these percentages are significantly lower for LAC (17%) and Africa (20%). Furthermore, roughly half of WRA in Africa (46%) and in LAC (52%) live in countries where abortion remains prohibited altogether (Category V) or is permitted when there is a risk to the pregnant person’s life (Category IV). By contrast, less than 1% of WRA live under such legal frameworks in Europe, whereas the proportion is over 20% in the Asia region.

**Table 2.** Percentage breakdown of women of reproductive age in each region by abortion legality category

	I	II	III	IV	V	VI (Varies)
Africa	9.04%	11.39%	33.19%	33.92%	12.46%	0.00%
Asia	38.15%	34.46%	7.11%	16.62%	3.66%	0.00%
Europe	85.31%	8.77%	5.84%	0.08%	0.01%	0.00%
LAC	16.60%	0.12%	11.35%	44.37%	7.79%	19.79%
Northern America	10.08%	0.00%	0.00%	0.00%	0.00%	89.92%
Oceania	69.12%	2.27%	1.27%	27.05%	0.29%	0.00%

Notes: (I) abortion permitted on request (gestational limits vary); (II) abortion permitted on broad social or economic grounds; (III) abortion permitted on health grounds (including risk to physical and mental health); (IV) abortion permitted to save the pregnant person’s life; (V) abortion prohibited altogether; and (VI) laws vary at state level (federal systems).

### Africa

**Figure 2.**
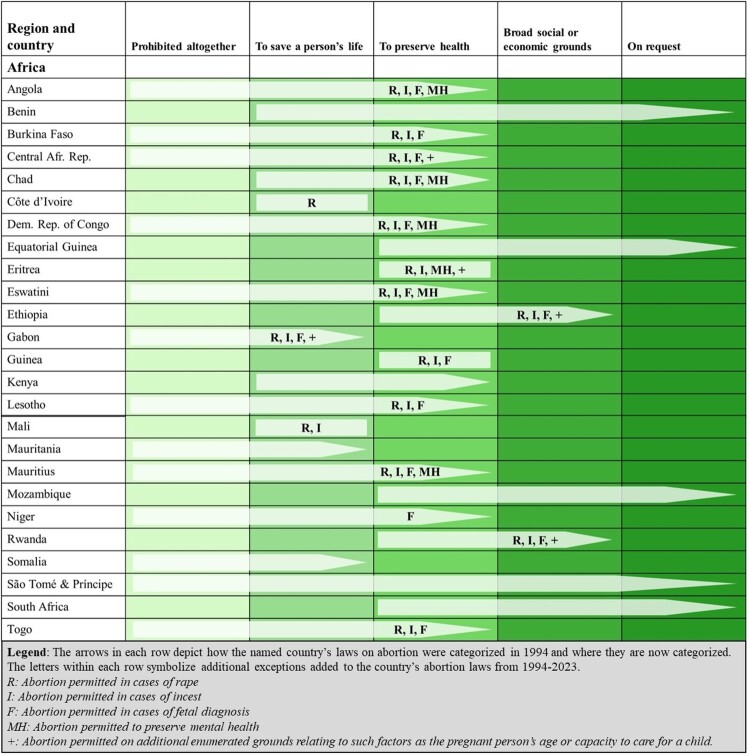
Liberalisations in countries’ abortion laws from 1994 to 2023 in the Africa region

Africa is the world’s largest region in number of countries and second-largest in population. This analysis encompasses 54 countries in Africa that are home to 18% of the world’s population of WRA. It is also the region that has had the greatest number of countries liberalise their abortion laws since 1994. As shown in Figure 2, in this time, 25 African states have liberalised their abortion laws, resulting in over 147 million WRA living under more liberal abortion laws than they would have 30 years ago. Notably, five of these countries – Chad, Benin, Central African Republic, Rwanda, and Angola – liberalised more than once, mostly to introduce enumerated grounds for legal abortion such as in cases of rape or incest. Furthermore, 13 states have reversed total bans on abortion. A handful of these liberalisations (South Africa, Benin, São Tomé & Príncipe, and Mozambique) have been to permit abortion on request, whereas almost half (12) have been to permit abortion where there is a threat to the pregnant person’s health.

Despite the high number of liberalisations, the legality of abortion remains significantly constrained. Nearly half of all WRA in Africa (46%) still live in countries in the two most restrictive categories (abortion banned altogether or permitted to preserve the person’s life). This includes the 12% of WRA who live in the region’s five countries where abortion is prohibited under all circumstances (representing the greatest absolute number of WRA in this category across all regions, at over 43 million). Indeed, Africa has the lowest proportion of WRA (9%) living in countries that permit abortion on request. Three countries (Ethiopia, Rwanda, and Zambia) making up 11% of WRA permit abortion on broad social and economic grounds, while 25 countries (33% of WRA) permit abortion to preserve the pregnant person’s health.

### Asia

**Figure 3.**
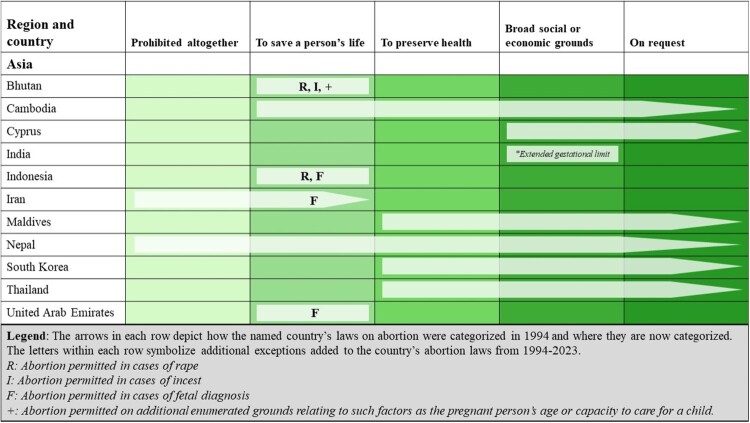
Liberalisations in countries’ abortion laws from 1994 to 2023 in the Asia region

Asia is the largest geographic region in terms of population. The countries included in our analysis are home to 60% of the world’s population of WRA spread across 50 different countries. As shown in Figure 3, 11 countries in this region have liberalised their abortion laws since 1994, resulting in 511 million WRA living under more liberal abortion laws than they would have 30 years ago. Of these 11 countries, 2 overturned absolute bans on abortion (Nepal and Iran) and 6 reformed their laws to permit abortion on request (Cambodia, Nepal, Maldives, Cyprus, Thailand, and South Korea).

Although 73% of WRA in this region live in countries where abortion is permitted on request or on broad social or economic grounds, Asia also has the greatest absolute number of WRA living in the two most restrictive categories: 237 million across 19 countries (20% of the regional total). Three countries (Laos, the Philippines, and Iraq, totalling 4% of WRA) ban abortion altogether, while 16 countries (17% of WRA), including several countries in the Middle East, narrowly permit abortion to preserve a person’s life. Seven countries (7% of WRA in the region) permit abortion on health grounds, while four countries (34% of WRA) allow abortion on broad socioeconomic grounds. Twenty countries, representing 38% of WRA in the region, permit abortion on request.

### Europe

**Figure 4.**
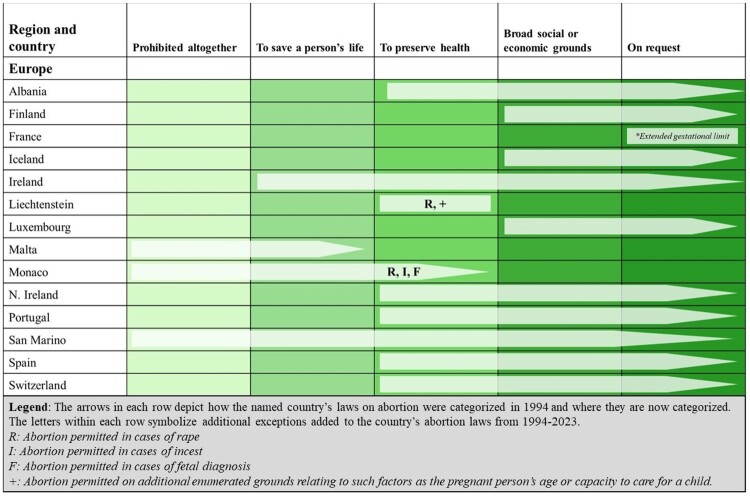
Liberalisations in countries’ abortion laws from 1994 to 2023 in the Europe region

Our analysis encompassed 45 European states, making up 8% of the world’s population of WRA. As shown in Figure 4, since 1994, 14 countries in this region have liberalised their abortion laws; overwhelmingly, these have been reforms to permit abortion on request (11 states). Currently, 85% of WRA in the region live in the 39 countries where abortion is permitted on request. Nearly 9% live in Great Britain, the only country where abortion is legal on broad social and economic grounds, while just under 6% live in the three countries (Liechtenstein, Monaco, and Poland) where abortion is permitted to preserve the pregnant person’s life or health.

There are three regional outliers: Andorra, Malta, and Poland. Andorra prohibits abortion under all circumstances, the only country in the region to remain in Category V. In 2023, Malta amended its absolute ban to permit abortion to preserve the life of the pregnant person, but with stringent requirements that the abortion be carried out by a medical team of at least three specialists including obstetricians or gynaecologists, as well as a specialist in the condition that is jeopardising the pregnant person’s life. Poland is also one of only four countries in the world to have removed legal grounds for abortion in the past 30 years. In 1996, Poland expanded its abortion law to encompass socioeconomic grounds, though this was invalidated the following year by its Constitutional Tribunal.^9^ Then, in 2020, the Constitutional Tribunal overturned an existing provision of Polish law permitting abortion in cases of fetal diagnoses. This decision sparked mass demonstrations in support of abortion rights. In 2023, the European Court of Human Rights recognised that the regressive restrictions resulting from the 2020 ruling of the Constitutional Tribunal, involving judges appointed by a severely irregular procedure that did not meet rule of law requirements, violated a woman’s right to private life.^10^ Notably, following the 2023 elections the Polish government has pledged to legalise abortion and the ongoing legislative process holds the potential for significant law reform.

### Latin America and the Caribbean

**Figure 5.**
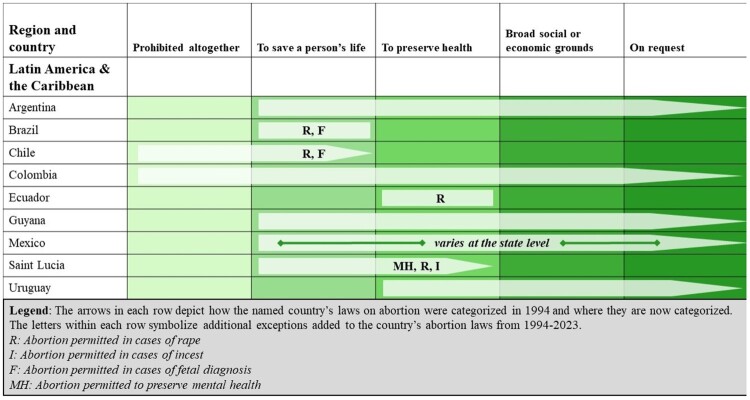
Liberalisations in countries’ abortion laws from 1994 to 2023 in the LAC region

Our analysis for Latin America and the Caribbean (LAC) included 34 countries, encompassing 9% of the world’s population of WRA. Since 1994, nine countries in the region have liberalised their abortion laws, resulting in 128 million WRA living under more liberal abortion laws than they would have 30 years ago (see Figure 5). This trend accelerated in recent years as a result of the Green Wave (“Marea Verde”) movement. Originating in Argentina, the movement secured its first victory in 2020 when Argentina enacted a new law permitting abortion on request. The following year, a decision from the Colombia Constitutional Court decriminalised abortion during the first 24 weeks of pregnancy, expanding on a 2006 ruling overturning Colombia’s absolute abortion ban and legalising abortion under certain circumstances. Later in 2021, the Supreme Court of Mexico issued a groundbreaking decision recognising a constitutional right to safe, legal, and free abortion services within a “short period” of time in early pregnancy, as well as on specific grounds thereafter. Due to Mexico’s federal system in which laws on abortion are determined at the state level, Mexican states are still in the process of reforming their laws to comply with the Supreme Court’s decision. A subsequent 2023 decision invalidated all federal criminal abortion provisions.

Although there has been notable progress in LAC, strict abortion laws remain in many countries. Over half of WRA live in countries where abortion is prohibited altogether (seven countries, 8% of WRA in the region) or permitted to preserve the person’s life (nine countries, 44% of WRA). Three countries, accounting for just 0.1% of WRA in the region – Barbados, Belize, and Saint Vincent & the Grenadines – permit abortion on social and economic grounds, while nine countries, home to 11% of WRA, permit abortion to preserve the person’s health. Finally, five countries (Argentina, Colombia, Cuba, Guyana, and Uruguay), representing 17% of the region’s WRA, permit abortion on request. Notably, Cuba was the only one of these countries to permit abortion on request prior to 1994.

In addition, LAC is home to two of the four countries to have removed legal grounds for abortion over the past thirty years: El Salvador and Nicaragua. In 1998, El Salvador revised its Penal Code, removing all legal grounds for abortion and instituting a blanket ban. In 2006, Nicaragua followed suit, introducing a law criminalising all abortion without exception. The impact of these retrogressions has been monumental: the 3.7 million WRA who call these countries home now live under laws which prohibit abortion in all circumstances.

### Northern America

Our analysis for Northern America consists of just two countries, Canada and the United States, which together make up 4% of WRA globally. While Canada has permitted abortion on request since 1988, the 2022 overturning of *Roe v. Wade* in the United States resulted in abortion being governed at the state level, representing a significant national and regional shift. This has led to a rapidly evolving legal landscape, with 14 states, home to 25% of the US population, effectively banning abortion by criminalising the provision of abortion services. At the same time, 17 states and the Capital District (Washington, DC) have further protected abortion rights in state law.

### Oceania

**Figure 6.**
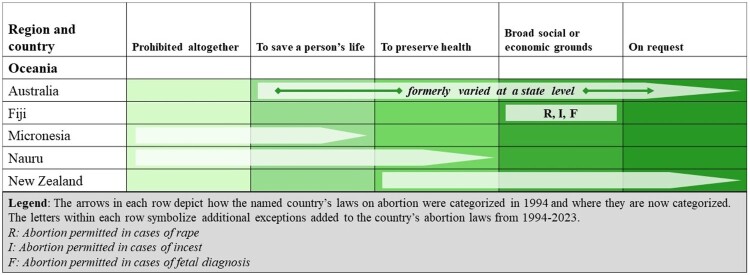
Liberalisations in countries’ abortion laws from 1994 to 2023 in the Oceania region

Consisting of 14 countries (Australia, New Zealand, and 12 Pacific Island States), our analysis for Oceania makes up 0.55% of WRA globally. As shown in Figure 6, 5 states in Oceania have liberalised their abortion laws in the past 30 years, meaning 7.6 million WRA are living under more liberal abortion laws than they would have been 30 years ago. Australia is distinct in that although it does not have an overarching federal abortion law, each state in Australia and the Australia Capital Territory has passed laws permitting abortion on request during the past thirty years. Nearly 70% of WRA in this region live in the two countries that permit abortion on request (Australia and New Zealand). One country, Fiji, permits abortion on broad social or economic grounds (2% of WRA), while three countries (Nauru, Samoa, and Vanuatu) permit abortion to preserve health, representing around 1% of WRA. Six countries allow abortion to preserve the pregnant person’s life, accounting for 27% of WRA in the region (in large part due to the population of Papua New Guinea). Just two countries (Tonga and Palau) prohibit abortion altogether, making up less than 1% of WRA.

## Discussion

There has been considerable progress towards the recognition of abortion rights in the 30 years since the International Conference on Population and Development. Such changes cannot be strictly attributed to ICPD and indeed are a culmination of strong civil society coalitions including gender justice movements, medical and public health organisations, and legal and human rights advocates, consistently elevating the concrete harms resulting from restrictive abortion laws and the impacts on individuals, families, and society. As part of this effort, political agreements and legal developments at the United Nations, alongside regional frameworks and mechanisms, have been critical in driving progress towards the recognition of abortion as both a human rights issue and a public health imperative. Although abortion remains contested in global political spaces, and therefore progress in these fora has been slow, there have been marked developments in both regional and international human rights law that have influenced reforms across the globe. United Nations Treaty Monitoring Bodies, which oversee states’ compliance with their respective human rights treaties, have nearly universally recognised the harms resulting from restrictive abortion laws and called on states to decriminalise abortion.^11,12^ Relatedly, in 2003, the African Union adopted the Maputo Protocol, its treaty on the rights of women, which is the first human rights instrument to recognise the right to abortion explicitly.^13^ This regional rights framework is likely a significant factor in the number and proportion of liberalisations the African region has seen in recent years. This human rights consensus is reinforced by global public health guidance, perhaps most significantly the World Health Organization’s recent Safe Abortion Guidelines, which call for full decriminalisation of abortion, recommend that states amend their laws to permit abortion on request, and recognise that individuals can safely self-manage abortion (meaning they can safely induce using medication abortion without clinical supervision) during the first trimester.^5^ The current guidelines build upon and strengthen the two previous editions of the guidelines, from 2003 and 2012, which have served as important entry points for ministries of health seeking to strengthen their policy frameworks and promote access to safe abortion care.^14,15^

The worldwide liberalisation of abortion laws has been accompanied by, and likely precipitated, significant reductions in maternal mortality and morbidity. While the number of maternal deaths resulting from unsafe abortion has decreased in line with the overall global reduction in maternal mortality, between 1990 and 2008 the proportion of abortion-related death was estimated to be the same: 13%.^16^ Since then, this may have declined further: the WHO estimates between 4.7% and 13.2% of all maternal deaths are now attributed to unsafe abortion.^5^ Despite this progress, 99.5% of deaths from unsafe abortion occur in developing regions, which are overwhelmingly where restrictive abortion laws remain.^17^ In resource-constrained settings, restrictive abortion laws have compounding impacts: people are more likely to experience unintended pregnancies and seek out unsafe abortions; they are, in turn, less likely to be able to access quality post-abortion care as health facilities are less accessible and under-resourced; and, where individuals can access post-abortion care, this exacerbates existing strains on healthcare systems.

It is critical to recognise that the 30 years of progress towards the liberalisation of abortion laws has not been uniform. The past five years have been marked by an increase in the number of liberalisations and by the fact that the majority of these reforms have permitted abortion on request. Since the beginning of 2019, 22 countries – over one-third of the total to have seen reform over the past 30 years – liberalised their abortion laws. Of these, 12 reformed their laws to permit abortion on request. There is a need to continue tracking global abortion laws to measure whether this accelerated rate of liberalisation holds.

It is also important to take a nuanced view in analysing these gains, as reforms that are more incremental in nature – while important – in practice, do not have the same effect on individuals’ ability to exercise reproductive autonomy as those that result in abortion being made legal on request. Indeed, where abortion is legal on limited grounds, lack of clarity on such grounds coupled with the continued use of criminal penalties, severe civil penalties, and the risk of professional sanctions can lead providers to refuse individuals abortion care in many contexts, including when their lives or health are at risk.^18,19^ Incorporating population data into analyses on abortion law reform is also important for conceptualising the potential scale that these reforms have on individuals’ lives. While all such reforms are important milestones of progress, the reality is that a greater absolute number of individuals are impacted by some liberalisations than others. Indeed, while this analysis has evidenced the trend towards liberalisation, it also highlights how many people still live in contexts with restrictive abortion laws: 25% of WRA, globally, live in countries where abortion is either not permitted under any circumstances or narrowly to preserve life.

Additionally, while legality of abortion is a useful proxy for measuring reproductive autonomy, a range of other factors impact whether individuals can safely access legal abortion services, even in contexts where abortion is broadly legal. There are countries, like South Africa, where abortion is permitted on request but unsafe abortion remains common due to stigma, refusals of providers and institutions to administer abortion care, and lack of knowledge about legal abortion.^20^ Additionally, restrictive policy measures such as bans on public funding for abortion, limitations on insurance coverage for abortion care, and lack of integration of abortion into public health systems also continue to undermine access in many contexts.^21^

At the same time, wider knowledge of and access to medical abortion has significantly changed the landscape by enabling people to safely self-manage abortion either independently, through remote (telemedical or online) support, or using accompaniment programmes created by feminist groups.^22^ Self-managed abortion is a particularly important option for those who are underserved by or untrusting of the formal healthcare sector, or who want greater privacy and/or control over their experience ending a pregnancy. Yet, despite WHO’s recognition that people can safely self-manage abortion in the first trimester, self-management remains illegal in most contexts, where criminal penalties also disproportionately affect the most marginalised or underserved.^5,23^

Even as developments in legality and provision have concretely expanded access to safe abortion care, relentless attacks on abortion continue in almost all contexts. The overturning of *Roe v. Wade* was a critical warning for abortion rights advocates worldwide that progress on abortion rights is constantly under threat. Similarly, the current regime in Argentina has vowed to overturn its landmark abortion law and China is taking steps towards imposing stricter gestational limits.^24,25^ Now more than ever, abortion rights advocates, public health actors, and governments must work together to safeguard the critical gains that have been made and continue accelerating progress.

## Conclusion

In the 30 years since ICPD, there has been significant progress towards the realisation of reproductive rights as countries across the globe have liberalised their abortion laws. The acceleration of this progress in the past five years – both in terms of the overall number of countries to have reformed their abortion laws and the marked shift towards liberalisation to permit abortion on request – is particularly notable. Continuing to track reforms to abortion laws worldwide will be essential for evaluating further progress and understanding the scale of law reforms on populations globally.

Yet, to guarantee true reproductive autonomy for individuals worldwide, more progress is urgently needed. Reproductive freedom requires that abortion laws are not only liberalised, but reconceptualised to prioritise individual autonomy and decision-making and to guarantee access to the full range of abortion options. States must invest in supporting those who wish to self-manage abortion to do so, while also strengthening formal healthcare systems to deliver safe abortion care. Further, abortion must be fully decriminalised so that individuals, abortion providers, and those supporting people having abortions can do so without fear of arrest, prosecution, and imprisonment. Finally, abortion cannot be approached in a vacuum; a holistic vision of reproductive freedom requires that all individuals have the resources to truly decide whether to carry a pregnancy to term, meaning that they have adequate educational opportunities, economic security, and social support systems to raise a family, if they so desire.

## Provenance statement

This article was not commissioned and went through external peer review.
